# Interventions to improve the well-being of family caregivers of patients on hemodialysis and peritoneal dialysis: a systematic review

**DOI:** 10.7717/peerj.11713

**Published:** 2021-07-20

**Authors:** Ana Carolina Hovadick, Viviane Rodrigues Jardim, Constança Paúl, Adriana Pagano, Ilka Reis, Heloisa Torres

**Affiliations:** 1 Escola de Enfermagem, Universidade Federal de Minas Gerais, Belo Horizonte, Minas Gerais, Brazil; 2 Instituto de Ciências Biomédicas Abel Salazar, Universidade de Porto, Porto, Portugal; 3 Faculdade de Letras, Universidade Federal de Minas Gerais, Belo Horizonte, Minas Gerais, Brazil; 4 Instituto de Ciências Exatas, Universidade Federal de Minas Gerais, Belo Horizonte, Minas Gerais, Brazil

**Keywords:** Family caregivers, Hemodialysis, Quality of life, Peritoneal dialysis, Well-being, Burden

## Abstract

**Background:**

The family caregivers of patients on hemodialysis (HD) and peritoneal dialysis (PD) typically experience higher burden than the general population because of the nature of tasks these caregivers need to carry out as a part of homecare. This fact influences both the caregivers’ quality of life and the quality of their care toward the patient. Thus, this study aimed to review the effectiveness and limitations of interventions in improving the well-being of family caregivers of patients on HD and PD.

**Methodology:**

A systematic review was performed according to the Preferred Reporting Items for Systematic Reviews and Meta-Analyses and the Cochrane Handbook for Systematic Reviews of Interventions (version 5.1.0). The Cochrane Library, Cumulative Index to Nursing and Allied Health Literature, Embase, MEDLINE, VHL Regional Portal, Scopus, and Web of Science databases were searched queried for randomized controlled trials that developed interventions aimed at improving the well-being of family caregivers of patients undergoing HD and/or PD from 2009 to 2020. The study protocol was registered at the International Prospective Register of Systematic Reviews (registration no. CRD42020151161).

**Results:**

Six studies met the inclusion criteria, all of which addressed caregivers of patients undergoing HD. All interventions reported in the included studies were carried out in group sessions, which addressed topics such as patient assistance and care, treatment complications, coping strategies, caregiver self-care practices, problem solving, and self-efficacy. The studies found significant improvement in the caregiver’s well-being.

**Conclusions:**

Group session interventions are effective in improving the well-being of family caregivers of patients undergoing HD. In regard to PD, there is insufficient evidence to make recommendations for caregivers of patients with this treatment.

## Introduction

Dialysis is prescribed when kidneys fail and are unable to remove waste and excess fluids from the body. Dialysis can be of two types: peritoneal dialysis (PD) and hemodialysis (HD). PD is more commonly carried out at home and consists of a cycle where a dialysate fluid is sent through a catheter to the peritoneal cavity of the patient and then removed. HD can be carried out either in a center or at home, where the patient’s blood goes through a filter called a dialyzer (Atif, 2016; Veguna Rani & Kamala, 2020).

Both treatments are time and effort demanding for both patients and family caregivers. Family caregivers are generally close friends or family members who provide free non-professional healthcare services to older people, people with illness, or people with disability (Family Caregiver Alliance, 2019). These caregivers play an essential role in assisting patients on HD and PD to carry on these burdensome and complex treatments, encompassing patients’ and caregivers’ mental, social, financial, and physical health.

The key points shared by caregivers of patients with other chronic diseases and caregivers of patients undergoing HD and PD are their homecare commitments (Wyld et al., 2021; Derrett et al., 2017). However, the latter group has major medical responsibilities and they play the integral role of the main care provider. Specific tasks of their role include special diet preparation, medication control, patient personal care (e.g., oral hygiene and bathing), and special attention with patients’ vascular/peritoneal access (Welch et al., 2014; Beanlands et al., 2005; Sinnakirouchenan & Holley, 2011). These caregivers also experience constant worry due to complications that patients may develop during treatment and face an inflexible therapy routine that affects their daily life. PD caregivers envisage even more stressful activities by assisting in therapeutic procedures, managing the medical supplies and constantly sterilizing the materials and home environment (Andreoli & Totoli, 2020; Favaro Ribeiro et al., 2009). All these activities, in addition to their personal demands, leave them in a state of distress and excessive burden. Consequently, caregivers of patients undergoing HD and PD have poorer QOL than the general population along with significant burden levels (Wyld et al., 2021; Derrett et al., 2017).

Such heavy workload and high levels of burden adversely affects the well-being of caregivers, making them more susceptible to depression, anxiety, and other medical conditions. This eventually leads to increased public and private healthcare expenditures (Cloutier et al., 2020). Furthermore, it impacts the care provided to the patients and consequently, the success of their treatment (Cloutier et al., 2020). Thus, interventions toward the improvement of their well-being are important to alleviate such a critical situation (Kang et al., 2019; Gilbertson et al., 2019; Jafari-Koulaee et al., 2020).

There is a lack of information regarding support interventions for caregivers of patients undergoing HD or PD in the literature. The last review that aimed to evaluate the support interventions for these caregivers was developed in 2008 (Tong, Sainsbury & Craig, 2008). Here, we intend to evaluate the state of the current literature targeting this population. Therefore, this systematic review aimed to (1) evaluate the effectiveness and limitations of interventions reported in the literature focusing on the well-being of family caregivers of patients undergoing HD or PD and (2) identify the most effective intervention to improve the well-being of these family caregivers.

## Survey Methodology

### Materials and Methods

This study is a systematic review of the literature. Before this study was started, the review methods were established in accordance with the instruments of the Preferred Reporting Items for Systematic Reviews and Meta-Analyses (PRISMA) (Online Resource, Table S1) and the Cochrane Handbook for Systematic Reviews of Interventions Version 5.1.0 (http://handbook-5-1.cochrane.org/) (Higgins & Green, 2011; Liberati et al., 2009).

To ensure search reliability, the review protocol was registered into the International Prospective Register of Systematic Reviews before data extraction was completed (registration no. CRD42020151161) (Sideri, Papageorgiou & Eliades, 2018).

### Data sources and search strategy

The study population was envisaged as caregivers of patients undergoing home or in-center HD or PD. We sought for interventions that focused on improving the well-being of this population. Well-being has varied definitions in the literature; in this study, we define well-being as a subjective state of physical, mental, emotional and social life satisfaction that is always in a dynamic change (Dodge et al., 2012).

A search for published studies was performed using the databases of the Cochrane Library, Cumulative Index to Nursing and Allied Health Literature, Embase, MEDLINE *via* PubMed, Regional Portal of the Virtual Library of Health (BVS-Brazil), Scopus, and Web of Science, comprising studies published from 2009 to 2020. We selected this period because an earlier review had already analyzed reports published until 2008 (Tong, Sainsbury & Craig, 2008). Hence, there was a need to update the results of the previous study. Furthermore, this period comprises literature from the last decade, which is the most recent body of Science. In addition, the reference lists of eligible studies, review articles, gray literature, and experts on the fields HD and PD were also referred.

The review was conducted between June and July 2020. The search strategy was designed to simultaneously retrieve studies that addressed “quality of life/burden”, “caregivers” and “hemodialysis/peritoneal dialysis”. The keywords used in the search strategy were selected from Descriptors in Health Sciences and Medical Subject Headings platforms. These queries were performed in Portuguese, English, and Spanish in the BVS-Brazil. In other cases, the queries were performed in English only.

The search strategies used in each database were properly compiled, as well as the restrictions that were applied (Online Resource, Table S2). The search results were imported into the reference manager software EndNote Online, where duplicate publications were found (Clarivate Analytics, 2019).

### Inclusion

The selection phase was independently performed by two autonomous reviewers (Ana Carolina Hovadick and Viviane Jardim). First, the retrieved articles were evaluated on the basis of their titles and abstracts. Then, all included studies were read in full to completely verify their eligibility. Disagreements among reviewers were resolved through consultations and input from a third author (Heloisa de Carvalho Torres).

The following inclusion criteria were adopted in our review: candidate studies should be (i) written in English, Portuguese, or Spanish, (ii) published between 2009 and 2020, and (iii) report an intervention that aimed to improve the well-being of family caregivers of patients undergoing HD or PD.

### Exclusion

Studies not classified as randomized controlled trials (RCTs) were excluded from the present review. This exclusion criterion is based on the fact that RCTs have a high level of scientific evidence, which increases the reliability of the results. In addition, we excluded publications that (i) were not related to the scope of the study, such as studies in which the population did not comprise family caregivers of patients undergoing HD and/or PD, (ii) were not written in one of the previously mentioned languages, and (iii) did not perform an intervention to improve the well-being of family caregivers. Data retrieved at this stage were properly compiled (Online Resource, Text S1).

### Data extraction

Data extraction was carried out based on the Consolidated Standards of Reporting Trials 2010 checklist, which is used to improve the reporting of RCTs (Table 1) (Schulz, Altman & Moher, 2010). This step was performed by two independent reviewers (Ana Carolina Hovadick and Viviane Jardim), and all disagreements were solved through discussions with a third author (Heloisa de Carvalho Torres).

**Table 1:** Main information of the studies included in the review.

Title/year	Sample size	Tools and approaches	Duration/frequency	Content of the sessions	Quest.*	Outcome
Evaluating the Effect of Family-Centered Intervention Program on Care Burden and Self-Efficacy of Hemodialysis Patient Caregivers Based on Social Cognitive Theory: a Randomized Clinical Trial Study—2020 (Rabiei et al., 2020)	70	The intervention group (*n* = 35) received an empowerment program that was held in group sessions with the assistance of the research team, including a nephrologist, a psychiatric nurse, and a hemodialysis nurse. Two rooms were assigned: an educational one and a Patient-family health room. A written description of the goals, content, and implementation timing was given to the participants in the first session. In the last session CDs and booklets were provided. The control group received routine training pamphlets and brochures during the study. Also, at the end of the study they received two general sessions, an educational booklet, and a CD. The researcher’s contact number was provided to participants for relevant consultation or enquiry.	One session per week for four consecutive weeks (each session lasted about 2 h)	Introduction of the program and familiarization between the participants; problem solving; self-efficacy improvement strategies for caregivers (self-esteem, a sense of competence, self-awareness skills such as knowledge about rights and values, attitudes and strengths, creativity and reinforcement of goal-setting skills, development of self-assessment skills, and self-confidence); psychological and spiritual benefits of care to increase caregivers’ positive expectancies and reduce their negative expectancies from patient care.	A two-section questionnaire with patients’ demographic characteristics and questions on care burden, negative outcomes expectancies, positive outcomes expectancies, and self-efficacy.	The intervention was statistically efficient decreasing care burden and negative outcomes expectancies between groups (*P* ≤ 0.05). Also, a statistically significant increase in the positive outcomes expectancies (*P* ≤ 0.05) and in the self-efficacy was found between groups (*P* < 0.05).
The Effect of Psycho-educational Intervention on the Caregiver burden among Caregivers of Hemodialysis Patients—2019 (Torabi, Maslak & Radfar, 2019)	105	Intervention group 1 (*n* = 35) received a workshop session, while intervention group 2 (*n* = 35) held group discussion sessions. Group 1 was subdivided into two groups and group 2 into three groups. A control group (*n* = 35) received routine trainings and training packages at the end of the study for acknowledgment of participation in research.	Intervention group 1 received six sessions for 2 h. Intervention group 2 received four sessions for 4 h. One session per week was held.	Group 1: more familiarity with the end-stage renal disease; principles of self-care, ways to increase self-confident; reducing stress and managing time. Group 2 had the same thematic as group 1 plus: improving carer skills and increasing communication skills.	ZBI and DQ.	After intervention statistically significant reduction of burden between intervention and control groups was found for individual, social, emotional and overall burden (*p* < 0.0001).
The Effect of a Family-Based Training Program on the Care Burden of Family Caregivers of Patients Undergoing Hemodialysis—2019 (Sotoudeh, Pahlavanzadeh & Alavi, 2019)	70	Caregivers in the intervention group (*n* = 35) received lectures, group discussions, practice, homework, and question and answer based on an educational booklet held by a researcher. Relaxation techniques were also conducted. At the beginning of each session, previous discussions and participants’ homework were reviewed and the sessions ended with questioning and group discussion. The control group (*n* = 35) received usual care plan and also two supervised session meetings were held to discuss their problems, feelings, and experiences.	Two sessions per week for four consecutive weeks (each session lasted about 90 min)	Knowledge and awareness about the end stage of kidney disease; maintenance and promotion of physical health and importance of self-care; effective communicative methods; methods of expressing emotions; coping skills for stress management; promoting family and social relationships; prayer therapy.	ZBS and DQ.	Statistically significant reduction of burden between intervention and control groups was found after intervention *(p* < 0.001).
Effect of Educational Program on the Burden of Family Caregivers of Hemodialysis Patients—2016 (Ashghali Farahani et al., 2016)	38	Intervention group (*n* = 38) was divided into five subgroups of 5–8 people. The sessions were held with short PowerPoint-based lectures, group discussion, question and answer, and role playing. Caregivers were provided with contact number of the researcher for relevant consultation or enquiry. Control group (*n* = 38) received routine care.	Two sessions per week for two consecutive weeks. (each session lasted about 60 min)	Greeting/explanation of rules and basic concepts; Home care of hemodialysis patients; Home care of hemodialysis patients; Hemodialysis complications and appropriate actions.	CBI and DQ.	Statistically significant reduction of burden between intervention and control groups was found after intervention (*p* < 0.001).
The effect of supportive educative program on the quality of life in family caregivers of hemodialysis patients.—2017 (Ghane et al., 2017)	38	The intervention group (*n* = 38) was divided into 5 subgroups of 6–8 people. Trainings sessions were delivered by an expert psychiatric nurse who was previously trained and tested. The caregivers received the researcher’s phone number in case of emergent questions. The control group (*n* = 38) received routine trainings and a booklet.	Three sessions per week for two consecutive weeks (each session lasted about 1 h).	Greeting/explanation of rules and basic concepts; Home care of hemodialysis patients; basic concepts of coping strategies; problem-focused coping strategies, and effective communication skills; strategies for anger management; and stress reduction and anger management strategies.	SF-36 and DQ.	Statistically significant improvement in quality of life between intervention and control groups was found after intervention (*p* < 0.001).
Effectiveness of Problem-Focused Coping Strategies on the Burden on Caregivers of Hemodialysis Patients—2016 (Ghane et al., 2016)	38	The intervention group (*n* = 38) was divided into 2 groups of 8 people. Sessions adopted short PowerPoint facilitated lectures, group discussion, a question and answer period, educational booklet and role playing. At the end of first session an educational handout was given to all participants so that they could exercise at home what was worked on. Trainings sessions were delivered by an expert psychiatric nurse previously trained. The control group (*n* = 38) received an educational booklet.	Two sessions per week for two consecutive weeks (each session lasted about 1 h).	Greeting, explaining the rules and basic concepts; Problem-focused coping strategies and effective communication skills; Strategies for anger management; Stress reduction and anger management strategies.	CBI and DQ.	Statistically significant reduction of burden between intervention and control groups was found after intervention (*p* < 0.001).

**Note:**

* Quest: questionnaires applied in the interventions; DQ: demographic questionnaire; ZBI: Zarit Burden Interview; ZBS: Zarit Burden Scale; SF-36: Short Form Health Survey; CBI: caregiver Burden Inventor.

### Quality assessment

The risk of bias of the studies was independently analyzed by two reviewers (Ana Carolina Hovadick and Viviane Jardim) using the Revised Cochrane risk-of-bias tool for randomized trials (RoB 2.0). Disagreements were solved by mutual agreement and consultations to the RoB 2.0 User’s Guide (Shea et al., 2017; Higgins et al., 2019). The methodology used to assess the quality of this systematic review was based on the A Measurement Tool to Assess Systematic Reviews (AMSTAR2) (Shea et al., 2017).

## Results

### Selection

**Figure 1:**
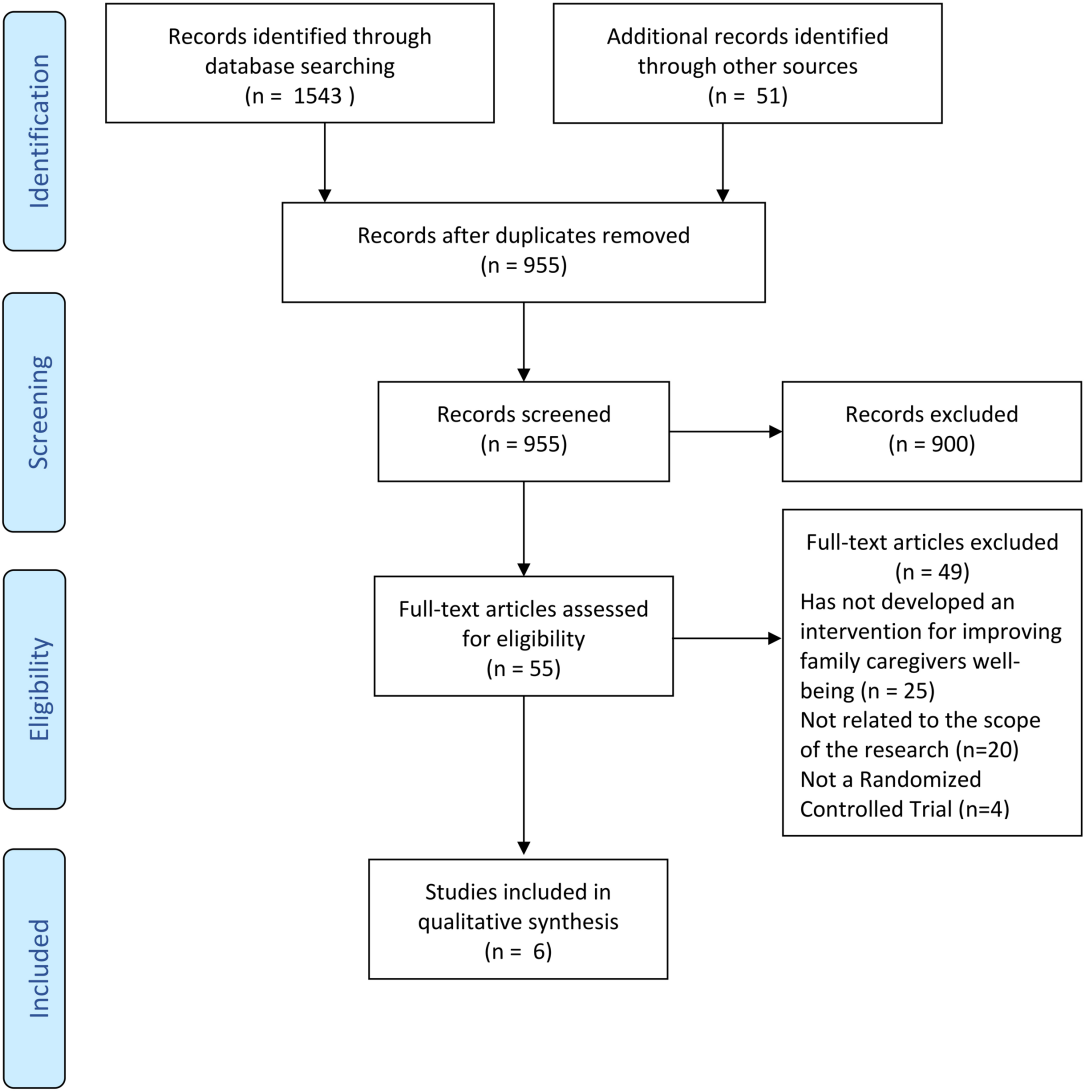
PRISMA flowchart diagram indicating the number of articles selected and excluded at each step.

A total of 1,543 potentially relevant publications were identified, among which, 639 duplicates were removed. Titles and abstracts of remaining publications were screened, and a total of 55 publications were selected to be fully read. Five studies were included in our review, and reference lists of included studies, review articles, and gray literature were assessed. In addition, experts in PD and HD were consulted on other remaining publications. Through this process, 51 articles were identified out of which one was included in the review. Thus, a total of six publications were eligible to proceed with data extraction and quality assessment. All steps were performed by two independent reviewers (Ana Carolina Hovadick and Viviane Jardim), and a third reviewer was consulted whenever disagreements arose (Heloisa de Carvalho Torres). To better illustrate the selection process, a PRISMA flowchart was developed, showing the number of publications selected at each step (Fig. 1).

### Study characteristics

Included studies focused on caregivers of patients undergoing HD. To the best of our knowledge, none of the studies meeting our inclusion criteria have targeted caregivers of patients undergoing PD. This indicates that there is either underreporting or absence of interventions for this population.

All studies developed the same intervention strategy: group session meetings. In this review, 1–8 groups of caregivers took part in these sessions, the sessions spanned from 2 to 6 weeks and the number of hours of intervention delivered to caregivers ranged from 4 to 16 h.

Coordinators of group sessions had different professional backgrounds. Three studies reported a nephrologist, a psychiatric nurse, and/or an HD nurse as the person responsible for delivering the content of the sessions (Ghane et al., 2016; Ghane et al., 2017; Rabiei et al., 2020). The remaining studies were conducted by the authors of the articles. All included studies were conducted in Iran.

### Tools, approaches and contents of each session

Although all interventions were conducted through group sessions, different tools and approaches were used to carry out each intervention, such as role playing, videos, pictures, booklets, question and answer time, workshop, homework, relaxation techniques, and short slide-based lectures. One study also adopted brainstorming techniques to improve group discussions (Table 1) (Torabi, Maslak & Radfar, 2019).

The contents discussed in the meetings were mainly about topics related to HD treatment, caregiver’s self-care, and self-efficacy improvement. Problem-focused coping strategies were implemented in all studies to improve proper communication, anger and stress management, and deep breathing. In addition, one study explored the psychological and spiritual benefits of care (Table 1) (Rabiei et al., 2020).

### Control groups

The control group received routine care in all studies. The majority of them (*n* = 4) also used booklets or pamphlets (Ghane et al., 2016; Rabiei et al., 2020; Sotoudeh, Pahlavanzadeh & Alavi, 2019; Ashghali Farahani et al., 2016). Three studies mentioned having validated these materials prior to use (Ghane et al., 2016; Ghane et al., 2017; Sotoudeh, Pahlavanzadeh & Alavi, 2019). Furthermore, after implementing the intervention with the intervention group, one study organized two supervised meetings with the control group, where participants could discuss their issues, feelings, and experiences (Sotoudeh, Pahlavanzadeh & Alavi, 2019). Another study provided the control group with training packages upon study completion to acknowledge patient participation in the research (Torabi, Maslak & Radfar, 2019). In the most recent study, in addition to routine care, upon conclusion the study participants also took part in two general sessions and received an educational booklet and a CD (Table 1) (Rabiei et al., 2020).

### Outcome of interventions

Well-being represents a subjective state of physical, mental, emotional, and social life satisfaction that is in constant dynamic change. In the included studies, the parameters used to measure the caregiver’s well-being were either QOL or levels of burden. All questionnaires were validated prior to use and evaluated similar topics on well-being as mental, physical, emotional, and/or social health. Statistical analyses used in each study were adequate and relevant. Besides descriptive statistics, chi-square, Fisher’s exact test, Mann–Whitney U test, repeated measures analysis of variance (ANOVA), two-way ANOVA, independent and paired t-test and t-couple were used.

### Burden

In five studies, burden was assessed using different questionnaires: Zarit Burden Interview, Zarit Burden Scale, and Copenhagen Burnout Inventory. One study developed and validated its own questionnaire. No significant differences in the mean burden scores were found between the intervention and control groups prior to the intervention. Likewise, no significant differences were found in the baseline demographic variables between the two groups. All studies reported statistically significant improvements in burden levels of caregivers in the intervention group compared with the control group (*p* < 0.05).

### Quality of life

Here we use a broad definition of QOL that includes economical, biological, psychological and social aspects of wellbeing (Ghane et al., 2016). None of the included studies provide a definition of quality of life. QOL was assessed in only one study using the SF-36 questionnaire. No significant differences in the mean QOL scores were found between the intervention and control groups prior to the intervention. Similarly, no significant differences were found in the baseline demographic variables between the two groups. The study concluded significant improvement of QOL between the groups (*p* < 0.001).

### Quality assessment

Bias analyzed through RoB 2.0 showed that all included studies have a low risk of bias in all five domains that the instrument evaluates: bias arising from the randomization process, bias due to deviation from intended interventions, bias due to missing outcome data, bias in outcome measurement, and bias in reporting results (Online Resource, Tables S3–S8).

All included studies obtained public funding for their development. Four of them declared no conflicts of interest (Ghane et al., 2017; Rabiei et al., 2020; Sotoudeh, Pahlavanzadeh & Alavi, 2019; Ashghali Farahani et al., 2016). The remaining studies did not include any statement regarding conflicts of interest (Ghane et al., 2016; Torabi, Maslak & Radfar, 2019).

The methodological quality of the present review was evaluated through the AMSTAR2 instrument checklist (Online Resource, Table S9). This evaluation revealed that the present review has moderate quality. The possible reasons were our failure to perform a meta-analysis given the small number of studies that met the inclusion criteria and the heterogeneity in the study design. To reinforce the methodological quality of this review, we provide the complete PRISMA checklist that was used as a guide for the development of the study (Online Resource, Table S1).

## Discussion

Despite extensive scientific literature that indicated low levels of well-being in family caregivers of patients undergoing HD and PD, as well as the need for interventions aimed at improving this issue, few relevant interventions have been developed (Tong, Sainsbury & Craig, 2008; Torabi, Maslak & Radfar, 2019; Belasco et al., 2006). Most studies have focused on the patients and their well-being without giving attention to the caregiver (Dudar et al., 2019; Gerasimoula et al., 2015; Alikari et al., 2015).

The last review that evaluated parameters similar to our study was carried out in 2008 (Table 2) (Tong, Sainsbury & Craig, 2008). Tong et al. reported the a lack of interventions to improve the well-being of caregivers of patients undergoing HD and PD. The absence of high-quality evidence of interventions to improve the well-being of these caregivers was also reported (Tong, Sainsbury & Craig, 2008). Since then, no reviews have aimed to provide an update on this situation. However, as reported herein, a small number of interventions have been developed. A comparison between the present review and the study by Tong et al. study shows that very little has changed in a decade with regard to this issue (Table 2). However, some improvements were made pertaining to the intervention type, tools and approaches and content, which were in partial agreement with the recommendations by Tong et al. As noted in the previous review, we reaffirm that interventions are important as “empowering methodologies” to actively involve participants in the construction of new interventions to promote their well-being. This methodology helps researchers find key points to be focused on the interventions and engages caregivers, which improves the chances of successful results (Tong, Sainsbury & Craig, 2008).

**Table 2:** Summary of the present study and progress made since the Tong, Sainsbury & Craig (2008) review.

Review	Number of studies	Experimental design	Setting	Treatment in focus	Intervention type	Tools and approaches	Content	Limitations	Effectiveness	Suggestions for future interventions
Tong, Sainsbury & Craig (2008)	3	Only non-RCTs found	Spain and India	HD, PD and kidney transplant	Educational	Booklets and oral and written information	**HD and PD:** knowledge about the treatment. **Kidney Transplant:** diet, immunosuppressive medication, secondary effects, protecting the graft, monitoring side-effects and importance of self-care.	Few studies found, only educational interventions and only non-RCT studies included.	Effective results. But insufficient evidence to confirm the benefit of the interventions and make recommendations.	Focus on participatory action research, using internet-based information, online support groups, psychological therapy and practical support.
This review	6	Only RCTs included	Iran	HD and PD*	Educational group sessions	CDs, booklets, lectures, group discussions, homework, question and answer time, relaxation techniques and role playing.	HD treatment, caregiver’s self-care, and self-efficacy improvement.	Insufficient evidence for PD patients, few studies found, only educational group sessions as intervention, all studies developed in the same country.	Interventions based on group sessions are effective to improve the well-being of family caregivers of patients on HD.	Multicomponent interventions focusing on both disease-related problems and caregivers’ personal demands, information and communication technologies (ICTs) and coping strategies.

**Note:**

* None of the studies meeting our inclusion criteria have targeted caregivers of patients undergoing PD.

Physical, emotional, mental, and social disorders have been increasingly observed in family caregivers. Psychological disorders, such as depression and anxiety, are often reported in the literature. In addition to difficulties encouraged in the management of stress and anger, sleep disorders, restrictions on social activities, and mental health deterioration have also been reported (Gilbertson et al., 2019; Tong, Sainsbury & Craig, 2008; Schulz, Altman & Moher, 2010; Shimoyama et al., 2003). Such disorders are due to increased workload, reduced leisure time, disruption of family relationships, and, in most cases, a decrease in self-care practices (Gilbertson et al., 2019). In addition, these conditions worsened when the patient and the caregiver lived in the same household (Acton, 2002). Therefore, interventions that focus on improving these underlying causes tend to have successful results (Acton, 2002; Belasco & Sesso, 2002).

In addition, published studies have shown a direct relationship between QOL levels of patients undergoing HD or PD and of their respective caregivers. Briefly, improvement in the well-being level of patients has similar effects on their caregivers. A 2016 study developed in Singapore confirmed this effect. In that study, an intervention was developed involving daily health care of patients undergoing HD in a healthcare center. The center offered conversation groups, physical therapy exercises, and music sessions, among other leisure activities. Although only patients received daily care, these sessions showed an improvement in the QOL for both patients and caregivers (Yu et al., 2016). In our review, most interventions focused on disease-related topics, and these studies presented positive results on the well-being of caregivers. This outcome supports the fact that more knowledge about dialysis promotes better patient care and achieves more accurate treatment results (Moreira et al., 2018; Khorami Markani et al., 2015).

Another effect was also reported in the literature, and it was related to the fact that when caregivers devote time to self-care practices, besides improving their own well-being, the health of the patients also improved. This is because greater well-being of caregiver results in better patient care (Khorami Markani et al., 2015).

All reviewed studies used coping strategies in their group sessions. Coping strategies help people positively deal with adverse problems. Some of the reported benefits are adequate communication skills, anger management skills, and deep-breathing exercises for relaxation. These strategies have also been used in interventions for caregivers of patients with other comorbidities, such as dementia, heart failure, and cancer, achieving good results in their well-being. Thus, coping strategies have been proven to be very effective. Since none of the studies in our review explored the effects of these strategies separately, but as a minor component in the intervention, they should be further explored to investigate their particular benefits for these caregivers (Chen et al., 2015; Porter et al., 2011).

In this review, the fact that all studies have developed a similar type of intervention is a concern. Although group sessions were effective and promoted better well-being for caregivers of patients undergoing HD, it is important to examine the effectiveness of other types of interventions on this population. This prevents us from stating a claim on the best type of intervention. However, multicomponent interventions targeting both disease-related problems and caregivers’ personal demands, are more likely to deliver meaningful results. Furthermore, they are expected to affect not only the caregiver–patient relationship but also lead to reductions in public and private healthcare expenditures, since it would prevent chronic kidney disease complications in patients and health problems in caregivers (Cloutier et al., 2020; Acton, 2002; Khorami Markani et al., 2015; Sorensen, Pinquart & Duberstein, 2002).

At present, promising interventions are those that include the use of information and communication technologies (ICTs). ICTs are technological tools that can be used to connect people for a common purpose. A systematic review published in 2014 presented beneficial effects of telehealth on the well-being of family carers of patients with dementia, cancer, stroke, heart disease, spinal cord injury, brain injury, mental illness, and chronic diseases in general. Among the technological resources employed were videoconferences, text messages, phone calls, and web-based information. The results showed enhanced mental and physical health, higher QOL levels, improved caregiving knowledge and skills, more social support, and improved coping skills (Chi & Demiris, 2015). Since interest in caregiver’s QOL of patients undergoing HD and PD is comparable with providing interest in caregivers’ QOL in other chronic diseases, ICT became a good candidate to be explored in future studies (Chi & Demiris, 2015; Alwan et al., 2011).

A relevant fact regarding our review is related with our original aim, which was to evaluate the effectiveness of interventions for the well-being of family caregivers of patients undergoing both HD and PD treatments. As stated, no RCTs have targeted caregivers of patients undergoing PD. Since HD and PD differ greatly from one another, no generalization can be made for both of them. However, the insights gathered from the studies herein reviewed may be used to asses prospective studies of intervention impact on caregivers of patients undergoing PD.

We were unable to perform a meta-analysis due to low number of studies and heterogeneity in the study design, which is a limitation of this study. Furthermore, only two studies used the same measures to evaluate the effectiveness of interventions, and numerically comparable metrics have not been found in the reviewed studies.

Other biases in the included articles were identified. From our final sample, two studies were performed by the same group of authors, authors of three publications are affiliated to one common university, authors of another two are also affiliated to one common university, and all studies were carried out in the same country, namely, Iran. These biases can possibly affect our discussion, since the intervention outcome can be influenced by and be restricted to the local population of that country. Therefore, studies with different populations are necessary to verify if the results reported for Iran are applicable to other populations. Furthermore, two of the reviewed studies did not include any statement about conflicts of interest. Missing statements about conflicts of interest influences the transparency of results as the impartiality of the authors cannot be asserted (Ferris & Fletcher, 2010).

## Conclusions

The results of this review suggest that interventions based on group sessions are effective in improving the well-being of family caregivers of patients on HD. The most effective intervention has not yet been established, as no type of intervention other than group sessions has been reported in the reviewed literature. As regards PD, there is insufficient evidence to make recommendations for caregivers of patients with this treatment.

## Supplemental Information

10.7717/peerj.11713/supp-1 Supplemental Information 1 Supplementary Material. (1) Preferred Reporting Items for Systematic Reviews and Meta-Analyses (PRISMA) checklist (Table S1); (2) Search strategy applied in each database (Table S2); (3) Not included articles (Text S1); (4) Revised Cochrane risk-of-bias tool for randomized trials (RoB 2.0) of each study (Table S3 to S8); A Measurement Tool to Assess Systematic Reviews (AMSTAR2) checklist (Table S9);

10.7717/peerj.11713/supp-2 Supplemental Information 2 PRISMA checklist.

10.7717/peerj.11713/supp-3 Supplemental Information 3 Systematic Review and/or Meta Analysis Rationale.

## Additional Information and Declarations

### Data Availability

The following information was supplied regarding data availability: Raw data are available in the Supplemental File.
