# Supplementary material for: Interventions to improve the well-being of family caregivers of patients on hemodialysis and peritoneal dialysis: a systematic review
Source: PeerJ. 2021 Jul 20;9:e11713. doi: 10.7717/peerj.11713 (PMC8300494; doi:10.7717/peerj.11713)
Supplement: Supplemental Information 2 [file peerj-09-11713-s002.docx]

**Table S1.** PRISMA checklist.

| **Section/topic** | **#** | **Checklist item** | **Reported on page^*^** |
| --- | --- | --- | --- |
| **TITLE** | | |  |
| Title | 1 | Identify the report as a systematic review, meta-analysis, or both. | 1 |
|  |  | “Interventions to improve the well-being of family caregivers of patients on hemodialysis and peritoneal dialysis: a systematic review” | |
| **ABSTRACT** | | |  |
| Structured summary | 2 | Provide a structured summary including, as applicable: background; objectives; data sources; study eligibility criteria, participants, and interventions; study appraisal and synthesis methods; results; limitations; conclusions and implications of key findings; systematic review registration number. | 2 |
|  |  | “Background. The family caregivers of patients on hemodialysis (HD) and peritoneal dialysis (PD) typically experience higher burden than the general population because of the nature of tasks these caregivers need to carry out as a part of homecare. This fact influences both the caregivers’ quality of life and the quality of their care toward the patient. Thus, this study aimed to review the effectiveness and limitations of interventions in improving the well-being of family caregivers of patients on HD and PD.  Methodology. A systematic review was performed according to the Preferred Reporting Items for Systematic Reviews and Meta-Analyses and the Cochrane Handbook for Systematic Reviews of Interventions Version 5.1.0. The Cochrane Library, Cumulative Index to Nursing and Allied Health Literature, Embase, MEDLINE, VHL Regional Portal, Scopus, and Web of Science databases were searched queried for randomized controlled trials that developed interventions aimed at improving the well-being of family caregivers of patients undergoing HD and/or PD from 2009 to 2020. The study protocol was registered at the International Prospective Register of Systematic Reviews (registration no. CRD42020151161).  Results. Six studies met the inclusion criteria, all of which addressed caregivers of patients undergoing HD. All interventions reported in the included studies were carried out in group sessions, which addressed topics such as patient assistance and care, treatment complications, coping strategies, caregiver self-care practices, problem solving, and self-efficacy. The studies found significant improvement in the caregiver’s well-being.  Conclusions. Group session interventions are effective in improving the well-being of family caregivers of patients undergoing HD. Regarding PD, there is insufficient evidence to make recommendations for caregivers of patients with this condition.” | |
| **INTRODUCTION** | | |  |
| Rationale | 3 | “Describe the rationale for the review in the context of what is already known. | 3,4 |
|  |  | “Such heavy workload and high levels of burden adversely affects the well-being of caregivers, making them more susceptible to depression, anxiety, and other medical conditions. This eventually leads to increased public and private healthcare expenditures.(11) Furthermore, it impacts the care provided to the patients and consequently, the success of their treatment.(11) Thus, interventions toward the improvement of their well-being are important to alleviate such a critical situation.(12-14)  There is a lack of information regarding support interventions for caregivers of patients undergoing HD or PD in the literature. The last review that aimed to evaluate the support interventions for these caregivers was developed in 2008.(15) Here, we intend to evaluate the state of the current literature targeting this population.” | |
| Objectives | 4 | Provide an explicit statement of questions being addressed with reference to participants, interventions, comparisons, outcomes, and study design (PICOS). | 4 |
|  |  | “Therefore, this systematic review aimed to 1) evaluate the effectiveness and limitations of interventions reported in the literature focusing on the well-being of family caregivers of patients undergoing HD or PD and 2) identify the most effective intervention to improve the well-being of these family caregivers.” | |
| **METHODS** | | |  |
| Protocol and registration | 5 | Indicate if a review protocol exists, if and where it can be accessed (e.g., Web address), and, if available, provide registration information including registration number. | 4 |
|  |  | “To ensure search reliability, the review protocol was registered into the International Prospective Register of Systematic Reviews before data extraction was completed (registration no. CRD42020151161).(18)” | |
| Eligibility criteria | 6 | Specify study characteristics (e.g., PICOS, length of follow-up) and report characteristics (e.g., years considered, language, publication status) used as criteria for eligibility, giving rationale. | 4,5 |
|  |  | “The study population was envisaged as caregivers of patients undergoing home or in-center HD or PD. We sought for interventions that focused on improving the well-being of this population. Well-being has varied definitions in the literature; in this study, we define well-being as a subjective state of physical, mental, emotional, and social life satisfaction that is always in a dynamic change.(19)”  “The following inclusion criteria were adopted in our review: candidate studies should be (i) written in English, Portuguese, or Spanish, (ii) published between 2009 and 2020, and (iii) report an intervention that aimed to improve the well-being of family caregivers of patients undergoing HD or PD.”  “We selected this period because an earlier review had already analyzed reports published until 2008. (15) Hence, there was a need to update the results of the previous study. Furthermore, this period comprises literature from the last decade, which is the most recent body of Science.”  “Studies not classified as randomized controlled trials (RCTs) were excluded from the present review. This exclusion criterion is based on the fact that RCTs have a high level of scientific evidence, which increases the reliability of the results. In addition, we excluded publications that (i) were not related to the scope of the study, such as studies in which the population did not comprise family caregivers of patients undergoing HD and/or PD, (ii) were not written in one of the previously mentioned languages, and (iii) did not perform an intervention to improve the well-being of family caregivers. Data retrieved at this stage were properly compiled (Online Resource, text S1).” | |
| Information sources | 7 | Describe all information sources (e.g., databases with dates of coverage, contact with study authors to identify additional studies) in the search and date last searched. | 4,5 |
|  |  | “A search for published studies was performed using the databases of the Cochrane Library, Cumulative Index to Nursing and Allied Health Literature, Embase, MEDLINE via PubMed, Regional Portal of the Virtual Library of Health (BVS-Brazil), Scopus, and Web of Science, comprising studies published from 2009 to 2020. We selected this period because an earlier review had already analyzed reports published until 2008. (15) Hence, there was a need to update the results of the previous study. Furthermore, this period comprises literature from the last decade, which is the most recent body of Science. In addition, the reference lists of eligible studies, review articles, gray literature, and experts on the fields HD and PD were also referred.”  “The review was conducted between June and July 2020.” | |
| Search | 8 | Present full electronic search strategy for at least one database, including any limits used, such that it could be repeated. | Supplementary Material – Table S1 |
|  |  | In the Supplementary Material (Table S1) is available the full electronic search strategy for each database. | |
| Study selection | 9 | State the process for selecting studies (i.e., screening, eligibility, included in systematic review, and, if applicable, included in the meta-analysis). | 6 |
|  |  | “A total of 1543 potentially relevant publications were identified, among which, 639 duplicates were removed. Titles and abstracts of remaining publications were screened, and a total of 55 publications were selected to be fully read. Five studies were included in our review, and reference lists of included studies, review articles, and gray literature were assessed. In addition, experts in PD and HD were consulted on other remaining publications. Through this process, 51 articles were identified out of which one was included in the review. Thus, a total of six publications were eligible to proceed with data extraction and quality assessment. All steps were performed by two independent reviewers (A.C.H. and V.J.), and a third reviewer was consulted whenever disagreements arose (H.C.T). To better illustrate the selection process, a PRISMA flowchart was developed, showing the number of publications selected at each step (Figure 1).” | |
| Data collection process | 10 | Describe method of data extraction from reports (e.g., piloted forms, independently, in duplicate) and any processes for obtaining and confirming data from investigators. | 6 |
|  |  | “Data extraction was carried out based on the Consolidated Standards of Reporting Trials 2010 checklist, which is used to improve the reporting of RCTs (Table 1).(21) This step was performed by two independent reviewers (A.C.H. and V.J.), and all disagreements were solved through discussions with a third author (H.C.T.).” | |
| Data items | 11 | List and define all variables for which data were sought (e.g., PICOS, funding sources) and any assumptions and simplifications made. | Table 1. |
| Risk of bias in individual studies | 12 | Describe methods used for assessing risk of bias of individual studies (including specification of whether this was done at the study or outcome level), and how this information is to be used in any data synthesis. | 6 |
|  |  | “The risk of bias of the studies was independently analyzed by two reviewers (A.C.H. and V.J.) using the Revised Cochrane risk-of-bias tool for randomized trials (RoB 2.0). Disagreements were solved by mutual agreement and consultations to the RoB 2.0 User's Guide.(22,23)The methodology used to assess the quality of this systematic review was based on the A Measurement Tool to Assess Systematic Reviews (AMSTAR2).(22)” | |
| Summary measures | 13 | State the principal summary measures (e.g., risk ratio, difference in means). | NA |
| Synthesis of results | 14 | Describe the methods of handling data and combining results of studies, if done, including measures of consistency (e.g., I^2^) for each meta-analysis. | NA |
| Risk of bias across studies | 15 | Specify any assessment of risk of bias that may affect the cumulative evidence (e.g., publication bias, selective reporting within studies). | NA |
| Additional analyses | 16 | Describe methods of additional analyses (e.g., sensitivity or subgroup analyses, meta-regression), if done, indicating which were pre-specified. | NA |
| **RESULTS** | | |  |
| Study selection | 17 | Give numbers of studies screened, assessed for eligibility, and included in the review, with reasons for exclusions at each stage, ideally with a flow diagram. | 6, Figure 1. |
|  |  | “A total of 1543 potentially relevant publications were identified, among which, 639 duplicates were removed. Titles and abstracts of remaining publications were screened, and a total of 55 publications were selected to be fully read. Five studies were included in our review, and reference lists of included studies, review articles, and gray literature were assessed. In addition, experts in PD and HD were consulted on other remaining publications. Through this process, 51 articles were identified out of which one was included in the review. Thus, a total of six publications were eligible to proceed with data extraction and quality assessment. All steps were performed by two independent reviewers (A.C.H. and V.J.), and a third reviewer was consulted whenever disagreements arose (H.C.T). To better illustrate the selection process, a PRISMA flowchart was developed, showing the number of publications selected at each step (Figure 1).”  In the Figure 1 is available the number of studies screened, assessed for eligibility, and included in the review, with reasons for exclusions at each stage. | |
| Study characteristics | 18 | For each study, present characteristics for which data were extracted (e.g., study size, PICOS, follow-up period) and provide the citations. | 6, 7, 8, Table 1. |
|  |  | “Included studies focused on caregivers of patients undergoing HD. To the best of our knowledge, none of the studies meeting our inclusion criteria have targeted caregivers of patients undergoing PD. This indicates that there is either underreporting or absence of interventions for this population.  All studies developed the same intervention strategy: group session meetings. In this review, 1–8 groups of caregivers took part in these sessions, the sessions spanned from 2 to 6 weeks and the number of hours of intervention delivered to caregivers ranged from 4 to 16 h.  Coordinators of group sessions had different professional backgrounds. Three studies reported a nephrologist, a psychiatric nurse, and/or an HD nurse as the person responsible for delivering the content of the sessions.(24-26) The remaining studies were conducted by the authors of the articles. All included studies were conducted in Iran.”  “Although all interventions were conducted through group sessions, different tools and approaches were used to carry out each intervention, such as role playing, videos, pictures, booklets, question and answer time, workshop, homework, relaxation techniques, and short slide-based lectures. One study also adopted brainstorming techniques to improve group discussions (Table 1).(27)  The contents discussed in the meetings were mainly about topics related to HD treatment, caregiver's self-care, and self-efficacy improvement. Problem-focused coping strategies were implemented in all studies to improve proper communication, anger and stress management, and deep breathing. In addition, one study explored the psychological and spiritual benefits of care (Table 1).(26)”  “The control group received routine care in all studies. The majority of them (n = 4) also used booklets or pamphlets.(24,26,28,29)Three studies mentioned having validated these materials prior to use.(24,25,28) Furthermore, after implementing the intervention with the intervention group, one study organized two supervised meetings with the control group, where participants could discuss their issues, feelings, and experiences (28). Another study provided the control group with training packages upon study completion to acknowledge patient participation in the research.(27) In the most recent study, in addition to routine care, upon conclusion the study participants also took part in two general sessions and received an educational booklet and a CD (Table 1).(26)”  “Well-being represents a subjective state of physical, mental, emotional, and social life satisfaction that is in constant dynamic change. In the included studies, the parameters used to measure the caregiver's well-being were either QOL or levels of burden. All questionnaires were validated prior to use and evaluated similar topics on well-being as mental, physical, emotional, and/or social health. Statistical analyses used in each study were adequate and relevant. Besides descriptive statistics, chi-square, Fisher's exact test, Mann–Whitney U test, repeated measures analysis of variance (ANOVA), two-way ANOVA, independent and paired t-test and t-couple were used.”  “In five studies, burden was assessed using different questionnaires: Zarit Burden Interview, Zarit Burden Scale, and Copenhagen Burnout Inventory. One study developed and validated its own questionnaire. No significant differences in the mean burden scores were found between the intervention and control groups prior to the intervention. Likewise, no significant differences were found in the baseline demographic variables between the two groups. All studies reported statistically significant improvements in burden levels of caregivers in the intervention group compared with the control group (p <0.05).”  “Here we use a broad definition of QOL that includes economical, biological, psychological and social aspects of wellbeing. (24) None of the included studies provide a definition of quality of life. QOL was assessed in only one study using the SF-36 questionnaire. No significant differences in the mean QOL scores were found between the intervention and control groups prior to the intervention. Similarly, no significant differences were found in the baseline demographic variables between the two groups. The study concluded significant improvement of QOL between the groups (p < 0.001).”  “Bias analyzed through RoB 2.0 showed that all included studies have a low risk of bias in all five domains that the instrument evaluates: bias arising from the randomization process, bias due to deviation from intended interventions, bias due to missing outcome data, bias in outcome measurement, and bias in reporting results (Online Resource, tables S3 to S8).  “All included studies obtained public funding for their development. Four of them declared no conflicts of interest.(25,26,28,29)The remaining studies did not include any statement regarding conflicts of interest.(24,27)”  In Table 1 is available all the characteristics extracted from the selected studies. | |
| Risk of bias within studies | 19 | Present data on risk of bias of each study and, if available, any outcome level assessment (see item 12). | 8, Supplementary Material – Table S3 to S8 |
|  |  | “Bias analyzed through RoB 2.0 showed that all included studies have a low risk of bias in all five domains that the instrument evaluates: bias arising from the randomization process, bias due to deviation from intended interventions, bias due to missing outcome data, bias in outcome measurement, and bias in reporting results (Online Resource, tables S3 to S8).”  In the Supplementary Material (Table S3 to S8) is available the risk of bias of each study. | |
| Results of individual studies | 20 | For all outcomes considered (benefits or harms), present, for each study: (a) simple summary data for each intervention group (b) effect estimates and confidence intervals, ideally with a forest plot. | NA |
| Synthesis of results | 21 | Present results of each meta-analysis done, including confidence intervals and measures of consistency. | NA |
| Risk of bias across studies | 22 | Present results of any assessment of risk of bias across studies (see Item 15). | NA |
| Additional analysis | 23 | Give results of additional analyses, if done (e.g., sensitivity or subgroup analyses, meta-regression [see Item 16]). | NA |
| **DISCUSSION** | | |  |
| Summary of evidence | 24 | Summarize the main findings including the strength of evidence for each main outcome; consider their relevance to key groups (e.g., healthcare providers, users, and policy makers). | 9,10,11 |
|  |  | “The last review that evaluated parameters similar to our study was carried out in 2008 (Table 2).(15) Tong et al. reported the a lack of interventions to improve the well-being of caregivers of patients undergoing HD and PD. The absence of high-quality evidence of interventions to improve the well-being of these caregivers was also reported.(15) Since then, no reviews have aimed to provide an update on this situation. However, as reported herein, a small number of interventions have been developed. A comparison between the present review and the study by Tong et al. study shows that very little has changed in a decade with regard to this issue (Table 2). However, some improvements were made pertaining to the intervention type, tools and approaches and content, which were in partial agreement with the recommendations by Tong et al.. As noted in the previous review, we reaffirm that interventions are important as “empowering methodologies” to actively involve participants in the construction of new interventions to promote their well-being. This methodology helps researchers find key points to be focused on the interventions and engages caregivers, which improves the chances of successful results.(15)”  “In addition, published studies have shown a direct relationship between QOL levels of patients undergoing HD or PD and of their respective caregivers. Briefly, improvement in the well-being level of patients has similar effects on their caregivers. A 2016 study developed in Singapore confirmed this effect. In that study, an intervention was developed involving daily health care of patients undergoing HD in a healthcare center. The center offered conversation groups, physical therapy exercises, and music sessions, among other leisure activities. Although only patients received daily care, these sessions showed an improvement in the QOL for both patients and caregivers.(37) In our review, most interventions focused on disease-related topics, and these studies presented positive results on the well-being of caregivers. This outcome supports the fact that more knowledge about dialysis promotes better patient care and achieves more accurate treatment results.(38,39)”  “Another effect was also reported in the literature, and it was related to the fact that when caregivers devote time to self-care practices, besides improving their own well-being, the health of the patients also improved. This is because greater well-being of caregiver results in better patient care.(39)”  “All reviewed studies used coping strategies in their group sessions. Coping strategies help people positively deal with adverse problems. Some of the reported benefits are adequate communication skills, anger management skills, and deep-breathing exercises for relaxation. These strategies have also been used in interventions for caregivers of patients with other comorbidities, such as dementia, heart failure, and cancer, achieving good results in their well-being. Thus, coping strategies have been proven to be very effective. Since none of the studies in our review explored the effects of these strategies separately, but as a minor component in the intervention, they should be further explored to investigate their particular benefits for these caregivers.(40,41)”  “In this review, the fact that all studies have developed a similar type of intervention is a concern. Although group sessions were effective and promoted better well-being for caregivers of patients undergoing HD, it is important to examine the effectiveness of other types of interventions on this population. This prevents us from stating a claim on the best type of intervention. However, multicomponent interventions targeting both disease-related problems and caregivers' personal demands, are more likely to deliver meaningful results. Furthermore, they are expected to affect not only the caregiver–patient relationship but also lead to reductions in public and private healthcare expenditures, since it would prevent chronic kidney disease complications in patients and health problems in caregivers.(11,35,39,42)”  “A relevant fact regarding our review is related with our original aim, which was to evaluate the effectiveness of interventions for the well-being of family caregivers of patients undergoing both HD and PD treatments. As stated, no RCTs have targeted caregivers of patients undergoing PD. Since HD and PD differ greatly from one another, no generalization can be made for both of them. However, the insights gathered from the studies herein reviewed may be used to asses prospective studies of intervention impact on caregivers of patients undergoing PD.” | |
| Limitations | 25 | Discuss limitations at study and outcome level (e.g., risk of bias), and at review-level (e.g., incomplete retrieval of identified research, reporting bias). | 11,12 |
|  |  | “A relevant fact regarding our review is related with our original aim, which was to evaluate the effectiveness of interventions for the well-being of family caregivers of patients undergoing both HD and PD treatments. As stated, no RCTs have targeted caregivers of patients undergoing PD. Since HD and PD differ greatly from one another, no generalization can be made for both of them. However, the insights gathered from the studies herein reviewed may be used to asses prospective studies of intervention impact on caregivers of patients undergoing PD.  We were unable to perform a meta-analysis due to low number of studies and heterogeneity in the study design, which is a limitation of this study. Furthermore, only two studies used the same measures to evaluate the effectiveness of interventions, and numerically comparable metrics have not been found in the reviewed studies.  Other biases in the included articles were identified. From our final sample, two studies were performed by the same group of authors, authors of three publications are affiliated to one common university, authors of another two are also affiliated to one common university, and all studies were carried out in the same country, namely, Iran. These biases can possibly affect our discussion, since the intervention outcome can be influenced by and be restricted to the local population of that country. Therefore, studies with different populations are necessary to verify if the results reported for Iran are applicable to other populations. Furthermore, two of the reviewed studies did not include any statement about conflicts of interest. Missing statements about conflicts of interest influences the transparency of results as the impartiality of the authors cannot be asserted.(45)” | |
| Conclusions | 26 | Provide a general interpretation of the results in the context of other evidence, and implications for future research. | 12 |
|  |  | “The results of this review suggest that interventions based on group sessions are effective in improving the well-being of family caregivers of patients on HD. The most effective intervention has not yet been established, as no type of intervention other than group sessions has been reported in the reviewed literature. As regards PD, there is insufficient evidence to make recommendations for caregivers of patients with this treatment.” | |
| **FUNDING** | | |  |
| Funding | 27 | Describe sources of funding for the systematic review and other support (e.g., supply of data); role of funders for the systematic review. | Provided to the submission system. |
|  |  | “This work was supported by the by the National Council for Scientific and Technological Development (CNPq, Conselho Nacional de Desenvolvimento Científico e Tecnológico) [No. 303250/2019].” | |
