# Supplementary material for: Interventions to improve the well-being of family caregivers of patients on hemodialysis and peritoneal dialysis: a systematic review
Source: PeerJ. 2021 Jul 20;9:e11713. doi: 10.7717/peerj.11713 (PMC8300494; doi:10.7717/peerj.11713)
Supplement: Supplemental Information 3 [file peerj-09-11713-s003.pdf]

Dear PeerJ readers,

This Systematic Review was conducted in order to fill the gap in the literature to evaluate the effectiveness and limitations of different types of interventions to improve the well-being of caregivers of patients undergoing hemodialysis and peritoneal dialysis. Although this topic is of wide societal and economical interest the last review that had evaluated similar parameters was published in 2008.

We also explain that given the low number of studies that met the inclusion criteria of the present review and as most studies did not used numerically comparable metrics, we were not allowed to perform a robust meta-analysis.

Regards,

Ana Carolina Hovadick

On behalf of all the co-authors
